# Non-Contact Plant Growth Measurement Method and System Based on Ubiquitous Sensor Network Technologies

**DOI:** 10.3390/s110404312

**Published:** 2011-04-13

**Authors:** Jinweon Suk, Seokhoon Kim, Intae Ryoo

**Affiliations:** Department of Computer Engineering, Kyung Hee University, Korea; E-Mails: sjw0176@unitel.co.kr (J.S.); kimsh@khu.ac.kr (S.K.)

**Keywords:** ubiquitous sensor network, non-contact, plant growth measurement, infrared sensor, thickening form

## Abstract

This paper proposes a non-contact plant growth measurement system using infrared sensors based on the ubiquitous sensor network (USN) technology. The proposed system measures plant growth parameters such as the stem radius of plants using real-time non-contact methods, and generates diameter, cross-sectional area and thickening form of plant stems using this measured data. Non-contact sensors have been used not to cause any damage to plants during measurement of the growth parameters. Once the growth parameters are measured, they are transmitted to a remote server using the sensor network technology and analyzed in the application program server. The analyzed data are then provided for administrators and a group of interested users. The proposed plant growth measurement system has been designed and implemented using fixed-type and rotary-type infrared sensor based measurement methods and devices. Finally, the system performance is compared and verified with the measurement data that have been obtained by practical field experiments.

## 1. Introduction

The term ‘ubiquitous’ can be used for the networking environment in which users get whatever information they want anytime and anywhere. In this ubiquitous paradigm, users want to gather, analyze, and control data automatically in real-time at a remote place. To implement this kind of ubiquitous space, the so-called USN technology is required. In this research, the USN is defined as a networking system that gathers surrounding environmental information (temperature, humidity, pollution level, crack propagation, growth information and so on) by locating and managing sensors in all the necessary places in real-time. The USN technology can be applied to almost all the fields in our life. Some important examples which offer outstanding convenience for us include real-time remote control for infrastructure, production, marketing, healthcare, welfare, defense, traffic, agriculture, forestry, and fisheries [1,2]. Although the agricultural and forestry sectors are lagging in terms of adopting advanced information communication technologies, there has been a focus recently on better forest administration, developing state-of-the-art agricultural technology, and dealing with various climatic conditions and global warming by using convergence technology. Therefore, in this paper, we analyze the problems of the existing plant growth measurement methods, and discuss a new plant growth measurement method and system.

In this paper, we propose a new real-time non-contact plant growth measurement system based on the USN technology to automate the plant growth measurement process and to solve the problem caused by existing measurement tools (tapelines, vernier calipers, contact sensors, *etc*.) damaging plants while measuring growth parameters. We also implement this system for a group of plants near a farmhouse and verify its performance by comparison with the measurement data obtained by practical field experiments. In Section 2, we introduce the plant growth process and discuss several issues of the existing plant growth measurement tools. The architecture and components of the proposed system to solve the problem generated by the existing plant growth measurement tools are presented in Section 3. We summarize the experimental results of the proposed system and discuss its performance in Section 4 and finally reach our conclusions in Section 5.

## 2. Plant Growth Measurement

### 2.1. Plant Growth and Growth Measurement

**Figure 1.**
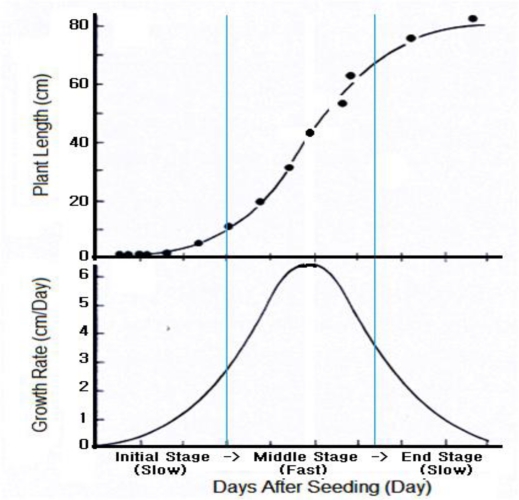
General plant growth characteristics.

The plant growth process is divided into three parts: growth, differentiation and development. The growth, which is a phenomenon involving an irreversible increase in size of tissues and organs as well as weight, is a quantitative change of the plant. The differentiation, which is an actual formation of tissues and organs is a qualitative change of the plant. The development is the growth and the differentiation of the plant body as time goes by. The overall plant growth stages can be summarized as follows: fertilization → gemmule → seed → germination → seeding → growth → flowering → fruit → aging. As seen in Figure 1, the general plant growth rate is represented by the curve known as Sigmoid curve that is classified into three stages: initial (slow) stage, middle (fast) stage and end (slow) stage. The initial stage is a period when plants establish their bodies after germination with the stored nutrients. In the middle stage, plants push out new shoots from the ground and complete their root systems. Biosynthesis and photosynthesis of leaves also occurs vigorously at that time. In the end stage, the plant growth rate slows down and there will be the competition for metabolites, water, light and mineral nutrients, as well as the accumulation of growth inhibitors [3].

**Figure 2.**
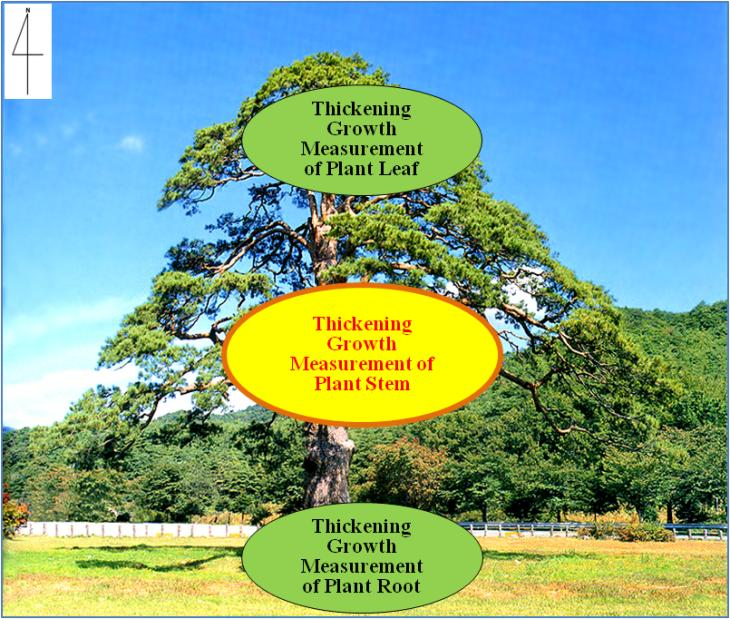
Plant growth measurement locations.

The growth characteristics of plants vary depending on the type of plant. Generally, plant growth measurements are taken for roots, stems, leaf area, leaf fat, stem cross section, leas section and so on. Figure 2 shows general plant growth measurement locations. The results of these measurements are used for assessing influences of climate, soil, nutrients, disease, and pests. Recently, measuring plant growth changes at a country level has become very important to look out for any climate changes. If we can precisely measure the plant growth per each growth cycle, we can make a detailed plant growth model and at the same time accurately predict and control the production of plants depending on environmental changes. Therefore, a real-time measurement system for the plant growth is very important in the fields of agriculture and forestry [4].

### 2.2. Plant Growth Measurement Tools and Devices

**Figure 3.**
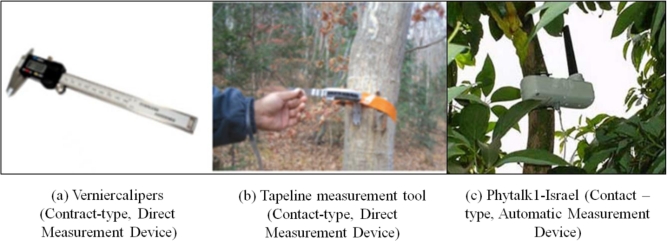
Existing plant growth measurement tools and devices.

The traditional measurement for plant growth has been mainly done by human eyes reading direct manual measurement devices (Figure 3(a,b)). In recent years, tactile sensors with the automatic data measurement and transmission features have been partially used (Figure 3(c)). However, with these manual measurement devices, we cannot guarantee the reliability of the measurement data because there could be wide variations in the measurement data depending on raters, measurement devices used and method of measurement. In addition, manual measurement devices like vernier calipers and contact-type devices may affect plant growth because they can injure and stress plants. Furthermore, there might also be many side effects caused by frequent or periodic measurements of the plant growth. Also, it is hard to measure the plant growth in a wide area simultaneously, especially in alpine regions or islands. The direct manual measurements definitely result in safety problems and cost increases [5–8].

## 3. Real-Time Non-Contact Plant Growth Measurement System Based on USN Technology

### 3.1. Ubiquitous Sensor Network (USN)

**Figure 4.**
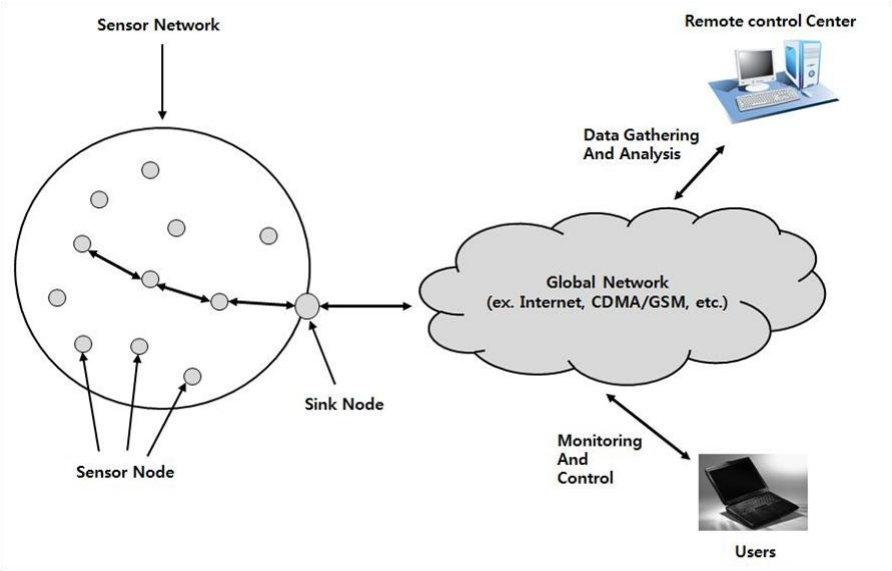
Basic system structure using USN technology.

In the USN environment, we install sensors for monitoring the status of remote forests and agricultural areas and connect them to a remote control center via a sink node which has a connection to the Internet. Figure 4 shows the basic system structure using USN technology. In the figure, sensor nodes send the sink node any measured data that have been obtained according to pre-configured conditions and service requests from the control center. The data received by the sink node are transferred and stored at the control center through global networks such as the Internet and CDMA networks. The data are then analyzed to determine the growth characteristics of plants, used as statistical data, and finally provided to any interested users [2,9,10].

### 3.2. Application of the Non-Contact Sensors for Plant Growth Measurement

The difference between contact and non-contact sensors is basically whether they are in contact with target plants or not. Contact sensors measure any change or displacement by bringing mechanical components like probes into contact with target plants. Examples of contact sensors include stereo comparators surface roughness measuring instruments, and limiter switches. Contact sensors have simpler structure compared with non-contact sensors, and have less of an environmental effect than these. Examples of non-contact sensors include infrared (IR) sensors, ultrasound sensors, and image sensors. These sensors use the light, supersonic, and electromagnetic characteristics for getting information such as capacitance, electromagnetic induction, and eddy current from the targets. Non-contact measurement is faster than contact-type measurement, causes no mechanical abrasion, and does not cause any damage to target plants. Therefore, they are used in the middle of any process or for any automatic production facilities [11,12].

**Figure 5.**
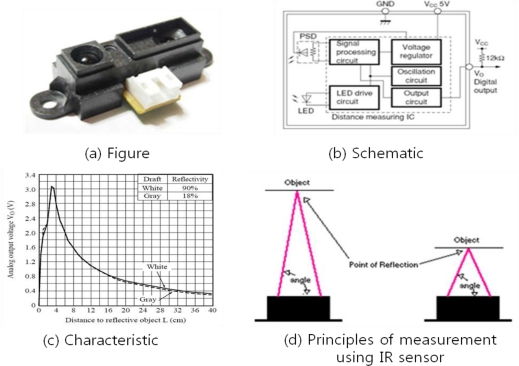
Infrared sensor used in this work (Model: SHARP GP2D120XJ00F Analog Output Type Short Distance Measuring Sensor).

To measure the plant growth by using non-contact IR sensors, we have to apply infrared radiation to the surface of a plant stem and convert the amount of it blocked into some quantity of electricity. This method, however, can only be applied to the case that the distance between the sensor and the target is less than 10 cm. As the sensors in our proposed plant measurement system are more than 10 cm away from the plants, we have to use another method which uses the angle of the reflected infrared energy to calculate the distance between the sensor and the target. The transmitting and receiving components of the IR sensors should be equipped with lenses for blocking any external disturbing lights by infrared filters. Figure 5 shows the IR sensor used in the proposed system, its schematic, output voltage characteristic by the distance to the reflective object, and principle of distance measurement. Also, Table 1 shows electro-optical characteristics of the sensor and Table 2 summarizes the absolute maximum ratings of sensor parameters [13].

**Figure 6.**
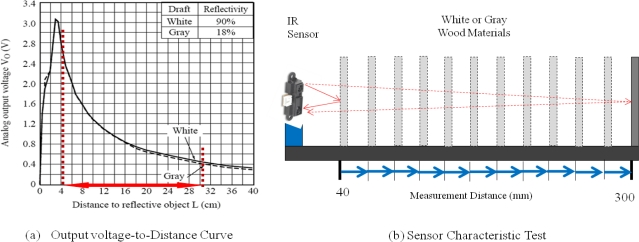
Output voltage-to-distance curve and sensor characteristic test.

In order to measure the thickening-growth of a plant, the distance between the plant and the sensor should first be obtained by using the analog output voltage of the IR sensors. That is, the voltage variations or changes of the IR sensors are converted into distance information, but voltage values of the IR sensors are not directly proportional to distance information. Furthermore, infrared rays are easily influenced by sunlight. To deal with this kind of measurement errors and reliability issues, we have done several hundred pre-tests of the IR sensor to get more reliable voltage-to-distance data conversions. Figure 6 shows the experimental environment. In the experiments, the IR sensor at a fixed location gets analog output voltages by moving a white or gray wood plate in one millimeter steps within the measurement distance range (40 mm ∼ 300 mm). Then, by comparing the sensor’s measurement distances with the real distances, we have found the sensor’s average measurement errors and determined the distance-adjustment values for every distance in the measurement range.

Table 3 shows some sample distance-adjustment values for the real distance range between 100 mm and 150 mm. As the name implies, the distance-adjustment values are used to compensate for the measurement errors when converting the sensor’s analog output voltage into a distance value. Distance compensation is simply achieved by adding the average adjustment value (AV) to the converted distance (CD). That is, the measured distances (MD) = CD + AV. This simple formula is used for the distance calculation program of the proposed non-contact plant growth measurement system to obtain the diameter of the target plant stem. From Table 3, the average adjustment value is the minimum when the distance between the sensor and the plant is 13.5 cm. Therefore, this distance is considered in the design and implementation of the proposed system. The measurement range and analog output voltage of the IR sensors used in the proposed system are 4 ∼ 30 cm and 0.4 ∼ 3.2 v, respectively.

### 3.3. A New Plant Thickening-Growth Measurement System Using the USN Technology

**Figure 7.**
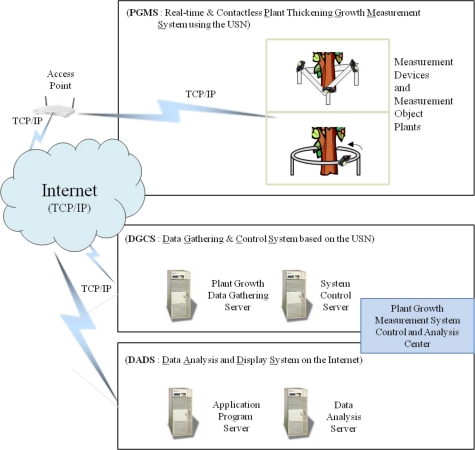
The proposed plant growth measurement system.

The proposed real-time non-contact plant thickening-growth measurement system is shown in Figure 7. The system consists of three sub-systems: plant growth measurement system (PGMS), data gathering and control system (DGCS), and data analysis and display system (DADS). The PGMS receives control commands from the control center, measures thickening growth of the corresponding plants, and sends the measured data to the plant growth data gathering server in the DGCS. The DGCS transmits data through the USN, stores the transmitted data and commands the PGMS to measure the plant growth. The DADS analyzes the measured data and provides the results to users in real-time. The proposed system measures plant stems by using non-contact sensors and provides plant growth information such as diameter, cross-sectional area, and thickening form. If we use the existing plant measurement methods, we can only measure the diameter of a plant. However, the proposed system enables us to estimate not only the diameter, but also the cross-sectional area and the thickening forms of plants at remote sites in real-time.

**Figure 8.**
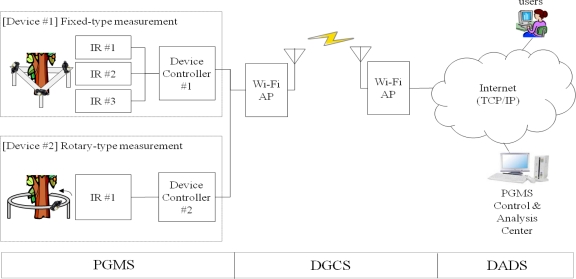
Simplified architecture of the proposed system.

**Figure 9.**
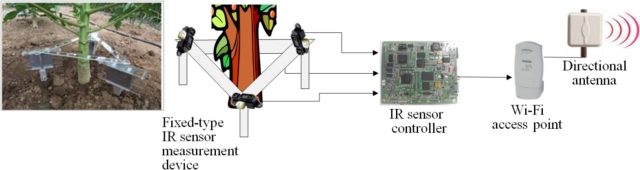
Fixed-type measurement method.

**Figure 10.**
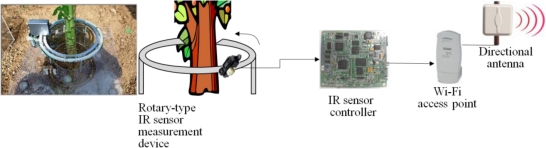
Rotary-type measurement method.

**Figure 11.**
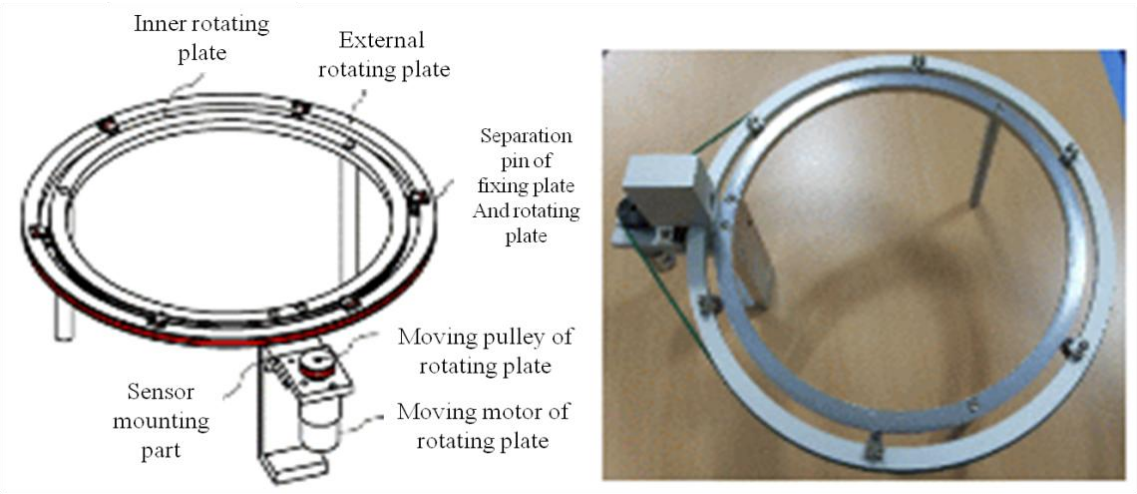
Rotary-type plant growth measurement device.

In this research, fixed and rotary measurement methods have been developed by using non-contact IR sensors in order to minimize the pre-described issues of the plant growth measurement systems as shown in Figure 8, where device #1 uses fixed IR sensors and device #2 uses a rotary IR sensor. More details of these two types of measurement methods are shown in Figures 9 and 10. Also, Figure 11 shows the 360° rotary-type plant growth measurement device that is designed and implemented in this research. This device is remotely controlled by the DGCS to measure the stem’s thickening-growth in a pre-defined measurement cycle. If the initial location (0°, azimuth) of the device is set due north, we can do a more detailed analysis on the growth status of the target plant by converting the measurement location into the azimuth angle. The sensors installed in the PGMS measure the growth parameters of the target plants and send the results to the DADS based on the USN technology in real-time. The USN technology-based DGCS sends the measured data from the sensor part of the PGMS to the server at the control center. Wi-Fi is used for the connection between the sensor nodes and the sink node, and access points (APs) is used for the connection between the sink node and local area network (LAN). Also, for optimizing experimental environment directional antenna (2.4 GHz, 13 dB, 35°) is installed for the place where buildings or obstacles are concentrated.

**Figure 12.**
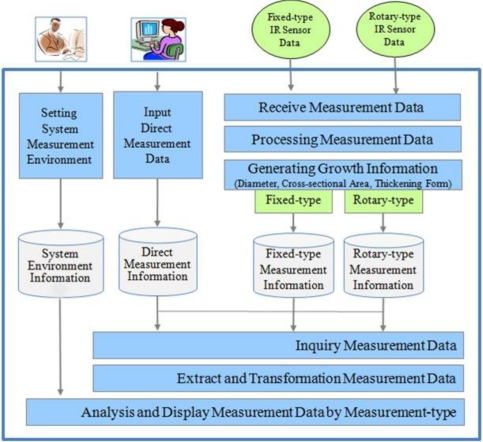
Software Architecture of the DADS.

Figure 12 shows the architecture of the data analysis and display system (DADS) which calculates the diameter and cross-sectional area of the plant stem and displays the corresponding thickening form. These growth parameters are then provided for interested group of users via the Internet.

**Figure 13.**
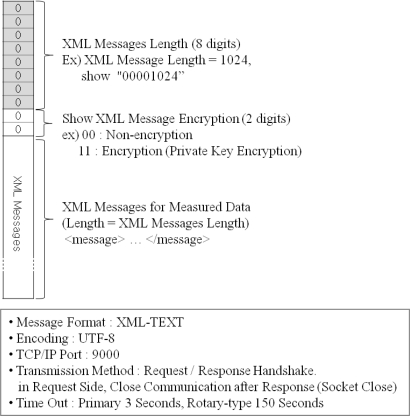
Packet structure for data transmission.

The data packet format used by the PGMS and the DADS is shown in Figure 13, and XML message types are shown in Table 4. Any measured data is encoded by UTF-8 and transferred by request/response handshaking method. Timeout timers are configured to be able to vary from 3 s to 150 s, depending on the type of sensors.

**Figure 14.**
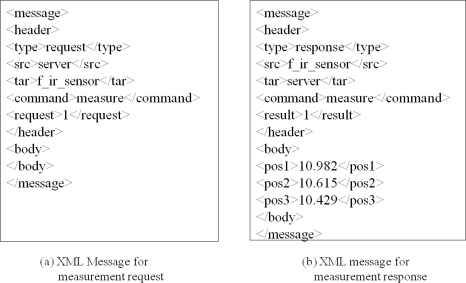
XML message examples for data transmission.

Figure 14 shows XML message examples for the data communication between the DGCS and the PGMS. Figure 14(a) is the XML message that is used by the server to ask the sensors to measure the plant growth, and Figure 14(b) is the XML message used by the sensors to send the measured data to the server. These messages are actually included in the protocol data unit shown in Figure 13.

**Figure 15.**
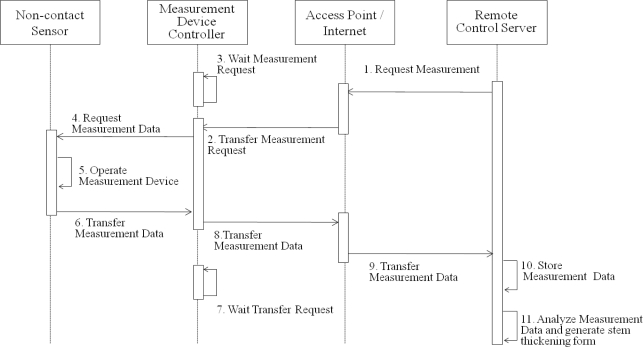
Data collection procedure of the plant growth measurement system.

Figure 15 shows a sequential diagram for the plant growth measurement and data processing. Firstly, the remote server sends control signals for measuring plant growth to the measurement devices. Once the control signals are received by the PGMS, the fixed-type and rotary-type non-contact sensors start to measure the stem’s thickening growth of the target plant. The measured data at the sensor nodes are converted into digital signals and sent to the remote servers through the sink node. Once the thickening-growth data is collected, it is also stored in the DADS. The measured data are then analyzed and provided for users. Figure 15 shows the overall sequential procedures of the proposed measurement system to get the plant’s thickening-growth form.

### 3.4. Generation of the Thickening-Growth Information Using the Measured Data

The DADS calculates the radius, diameter, circumference, and cross-sectional area of the target plant by using the collected growth data. In particular, the proposed system can generate the stem’s thickening-growth form by applying the LineDDA algorithm and 2-dimensional polar coordinate-to-circular coordinate transforms, which has not been possible with the existing measurements tools and methods.

**Figure 16.**
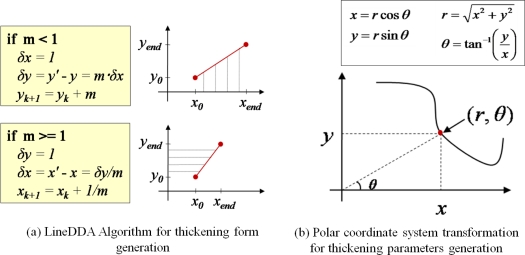
Line digital differential analyzer (LineDDA) algorithm and polar coordinate system transformation.

As described in Section 3.2, in order to generate the plant growth information, the distance between the plant and the sensor should be first obtained by using the distance adjustment formula; MD = CD + AV. Note that the MD is the average measured distance between the sensor and the target plant. If we subtract the MD from the distance between the sensor and central point (r), we can get a stem’s average radius (ȓ_i_) at the corresponding measurement point. Also, by calculating the average of the average radii at all the measurement points, we can get the stem’s average radius (ȓ) of the target plant. Meanwhile, in order for calculating the cross-sectional area and the thickening-growth form of the plant stem that has different radii, 2-dimensional polar coordinate system transformation can be used. Polar coordinates can easily be transformed to circular coordinates, which enables us to get the cross-sectional area and form of the plant stem. Figure 16 shows the principle and the formula of coordinate transformation between plane coordinate (*x*, *y*) and polar coordinate (*r*, *θ*) as well as the LineDDA algorithm.

There are several ways to calculate the cross-sectional area by using the polar coordinate system. The first method is to pre-calculate the area of one pixel on the polar coordinate and then multiply this by the number of pixels from the measured data. Although this method is useful for a fixed image, it is hard to make generalizations about the case that has any measured data. The second method is to calculate the average radius from many different radii and then use this for calculating the cross-sectional area of the target. The measurement error of this method is tolerable if the thickening form of the target is close to a circle. But, in case that the target has an indented thickening form, the measurement error increases accordingly. The third method is based on the characteristic that any line segment for the measured data on the polar coordinate is identical to the circumference of the circular coordinate. Therefore, we can obtain the cross-sectional area simply by substituting the length of line segment to the formula for calculating the area of the circle [14]. The proposed system uses the third method to calculate the cross-sectional area of the target plant stem as it has a minimum error compared to other methods and is easy to apply to the measured data from the PGMS. The thickening form of the target plant is displayed by transforming the polar coordinate into circular coordinate again.

**Figure 17.**
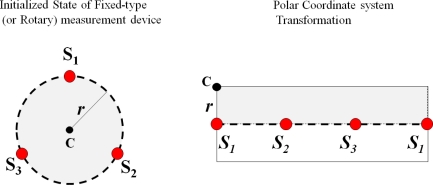
Generation of thickening form of the target plant (initial state).

**Figure 18.**
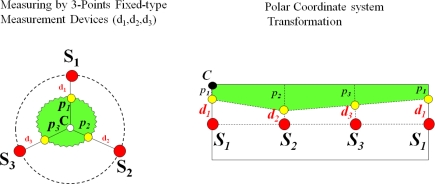
Generation of thickening form of the target plant (conversion to polar coordinate system).

**Figure 19.**
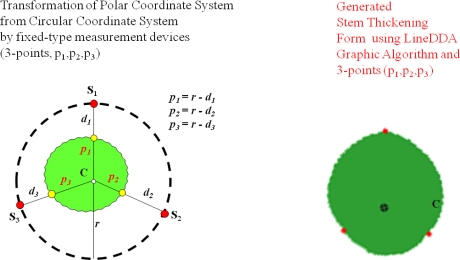
Generation of thickening form of the target plant (fixed-type).

**Figure 20.**
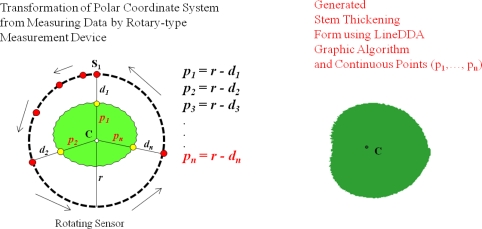
Generation of thickening form of the target plant (rotary-type).

In order to apply the above-mentioned algorithm and method to the data that will be measured by non-contact IR sensors and calculating the cross-sectional area of the plant stem, we have installed three sensors around the target plant for the fixed-type measurement. The sensors are equally spaced and the target plant is centered from these sensors. Figure 17 shows the polar coordinate system transformation with three IR sensors around the target plant. As shown in this figure, from the circle that is formed by three sensors, we can get four polar coordinate points. The next step is to connect the line segments by using the well-known line digital differential analyzer (LineDDA) algorithm (Figure 16(a)). After completing this, we can get the thickening form of the target plant as shown in Figures 18, 19 and 20, and can also easily get the circumference and cross-sectional area.

**Figure 21.**
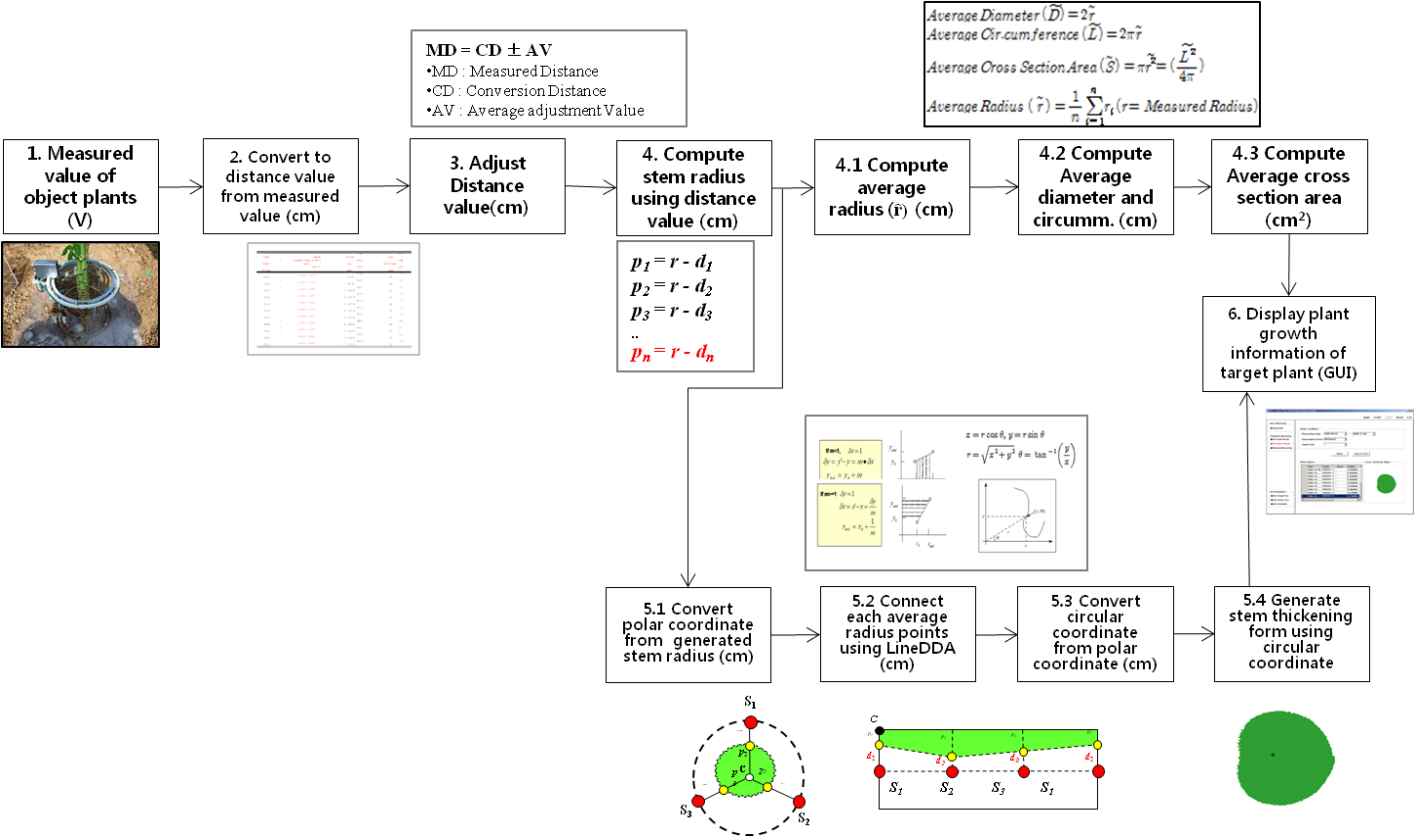
Process of generation of the plant growth information using the measured data.

The fixed-type measurement method may have intolerable errors in the calculation of cross-sectional area of the plant as it uses only four polar coordinate points. Theoretically, if we were to install an infinite number of sensors around the target plant, there would be no measurement and calculation errors! As a practical solution for this problem, we have developed the rotary-type measurement system to minimize the measurement errors as well as to measure the plant from all directions at pre-defined intervals. Figure 21 shows all the above-described growth information generation processes step-by-step.

## 4. Performance Evaluation

### 4.1. Implementation and Experimental Environment

**Figure 22.**
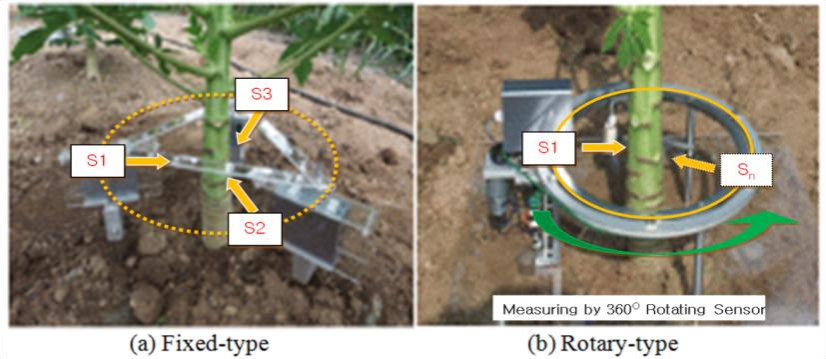
Field Test Environments (fixed-type and rotary-type).

The proposed system has been implemented with Microsoft Windows 2003 Server and SQL Server 2005 DBMS, and the application software has been developed using .NET. The client is implemented with Windows Vista on a laptop computer. The field test environment for measuring the plant growth is shown in Figure 22. In the figure, the device on the left side is the fixed-type measurement device using three IR sensors, and the device on the right side is the rotary-type measurement device which rotates one sensor through 360 degrees around the target plant. These two devices are remotely controlled by the DGCS as described in the last section. The measurements are automatically performed at the pre-configured time periods. The measured data are also transferred to the server through the USN and the Internet, and stored in the database according to each of measurement methods.

**Figure 23.**
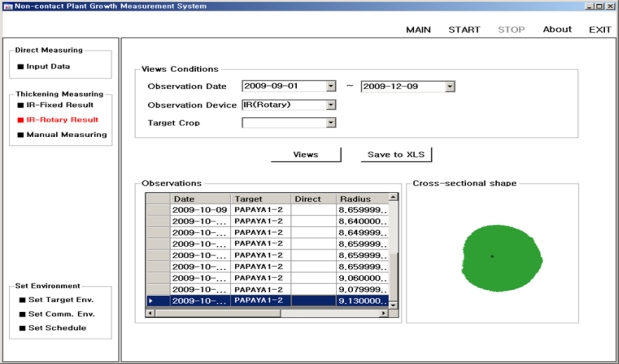
GUI of the Growth Measurement System.

Figure 23 shows a graphical user interface (GUI) screenshot of the non-contact plant growth measurement system implemented at the plant growth measurement, monitoring, and control center. From the menu buttons on the left hand side of this GUI, we can choose the type of sensor and set the test environment. The duration of measurement and type of device can also be chosen from the ‘Views Conditions’ menu box in this figure. According to the test conditions that are set by this GUI, the remote sensors at the PGMS measures the target plants. The measurement results are then transferred to the DGCS and analyzed by the DADS to display the growth parameters as shown in the “Observations” and “Cross-sectional shape” menu boxes of Figure 23.

### 4.2. Experimental Results and Analysis

Table 5 shows the data samples that are measured by the rotary-type measurement device and then converted into the measured distances (MDs) at the DADS. The rotary-type plant growth measurement device can get 130 data points at a time by setting the sensor’s rotation measurement deviation to 2.5 degrees.

**Figure 24.**
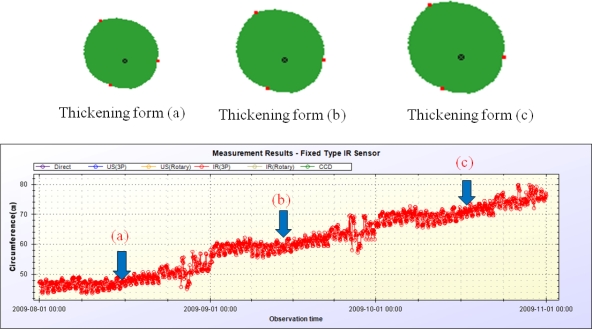
Measurement results obtained using the fixed-type measurement device.

**Figure 25.**
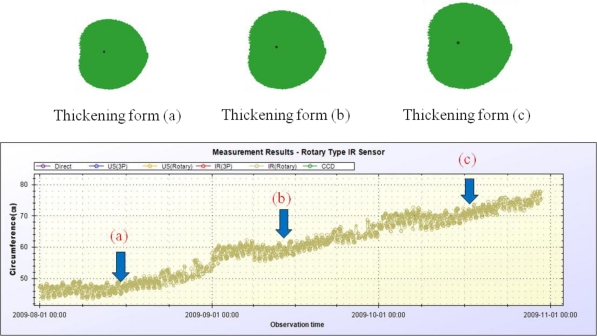
Measurement results obtained using the rotary-type measurement device.

Columns in this table show the measurement ID, target plant name, the measured data at 130 different measurement points, average cross-sectional area, average radius, and circumference of the target plant from left to right. By using these data and plant growth measurement method described in Section 3.4, we can generate the stem’s thickening forms. Figures 24 and 25 show the growth measurement results for the target plant (a papaya tree) by using the fix-type IR sensors and rotary-type IR sensor. These papaya seedlings are planted in April of 2009 and measured at the pre-configured times every day from August 2009 to October 2009.

The upper parts of these two figures show the thickening forms of the corresponding papayas. Time interval between thickening forms (a), (b), and (c) is one month. The thickening forms shown in the figures are automatically created by the DADS by using the measured data. Although, in the figures, there are only three thickening forms per each type of device for lack of space, it is possible to display continuous visible changes of thickening-growth process of the target plant with the DADS. Points on the circumference in Figure 24 correspond to the sensing points on the stem’s surface, and the point inside the circumference denotes the center mark of the sensors. The lower parts of Figures 24 and 25 show all the measurement results for the circumference during the same period. With this data, we can get a detailed growth rate of the target plant on daily basis.

The plant growth measurement results by using the proposed system are understandable over all, but have abnormal results at some time points. That is, at some measurement points, the circumference is less than that measured in the past. These measurement errors are presumed to be due to the presence of foreign debris on the stem’s surface which results in sensing errors. If we compare the measurement results of the rotary-type IR sensor with those of the fixed-type IR sensors, we could see more detailed thickening forms in case of using the rotary-type sensor. Measurement errors are also decreased with the rotary-type measurement method.

**Figure 26.**
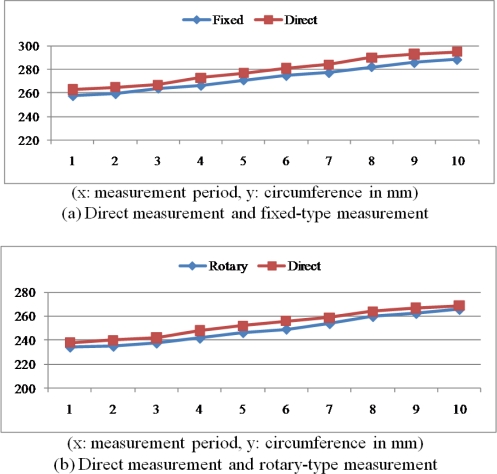
Comparisons of measurement methods.

As it is difficult to implement the fixed-type and rotary-type measurement methods for the same plant at the same time, we have combined the direct (or manual) measurement with each of these methods for performance comparison. Table 6(a) summarizes the measurement results for the same plant by using direct measurement tool and the fix-type IR sensors, and Table 6(b) summarizes the measurement results by using direct measurement tool and the rotary-type IR sensor for the same plant. For more obvious distinctions regarding the performance comparisons, Figure 26(a,b) shows the graphical presentations of the results in Table 6(a,b), respectively.

Table 7 compares and analyzes the qualitative performance items of the existing methods and the proposed methods. According to these comparison results, the proposed methods have been verified to outperform the existing methods in almost all of performance features. In particular, the proposed system makes it possible to measure uneven surfaces, display thickening forms, and perform remote measurement in real-time. Any research to improve the proposed USN-technology-based non-contact plant measurement method and system is for further study.

## 5. Conclusions and Remarks

This paper proposes the a USN technology-based real-time non-contact plant growth measurement system using non-contact IR sensors to solve the existing problems of plant measurement techniques. The proposed system has been implemented and then installed on plants to measure the plant growth parameters such as diameter, cross-sectional area and thickening form. Through the field tests, it has been verified that the proposed system can be used in the field of plant growth measurement. The proposed system makes it possible to measure uneven surfaces, display thickening forms, and perform remote measurements in real-time, which is nearly impossible with existing measurement methods.

However, for functionality, stability and accuracy, the proposed system needs to be continuously improved. The convenience and durability for the measurement parts of the rotary-type measurement system should be supported more. In order to minimize the measurement errors, we need to attempt to use ultrasonic, image, or LASER sensors rather than IR sensors. The measurement and estimation algorithms, including image processing techniques, should also be enhanced and optimized to get more accurate plant growth information. Finally, the proposed system needs to be upgraded to a plant growth model analysis system which is to be connected with global positioning system (GPS) and automatic weather system (AWS).

## Figures and Tables

**Table 1.** Electro-optical characteristics.

**Parameters**	**Conditions**	**MIN.**	**TYP.**	**MAX.**
Measuring distance range (cm)	reflective object (White paper)	4	-	30
Output terminal voltage (V)	L = 30 cm	0.25	0.4	0.55
Output voltage difference (V)	Output change at L change (30 cm 4 cm)	1.95	2.25	2.55
Average supply current (mA)	L = 30 cm	-	33	50

L: Distance to reflective object.

**Table 2.** Absolute maximum ratings.

**Parameters**	**Ratings**
Supply voltage (V)	−0.3 to +7
Output terminal voltage (V)	−0.3 to Vcc+0.3
Operating temperature (°C)	−10 to +60
Storage temperature (°C)	−40 to +70

**Table 3.** Average adjustment values of IR sensors (100 ∼ 150 mm distance range).

**Actual Distance (AD, mm)**	**Output Terminal Voltage of the Sensor (OS, V)**	**Converted Distance (CD, mm)**	**Average Adjustment Value (AV, mm)**
100.00	1.3318 ∼ 1.3347	94.38 ∼ 94.44	**+**5.59
105.00	1.2703 ∼ 1.2705	98.51 ∼ 98.78	**+**6.36
110.00	1.1957 ∼ 1.1959	106.25 ∼ 106.51	**+**3.62
115.00	1.1398 ∼ 1.1399	112.39 ∼ 112.79	**+**2.41
120.00	1.1008 ∼ 1.1011	117.34 ∼ 117.74	**+**2.46
125.00	1.0428 ∼ 1.0438	124.38 ∼ 124.71	**+**0.45
130.00	1.0048 ∼ 1.0050	129.15 ∼ 129.55	**+**0.65
**135.00**	0.9575 ∼ 0.9592	**135.16 ∼ 135.31**	**−0.24**
140.00	0.9092 ∼ 0.9097	141.12 ∼ 143.12	−2.12
145.00	0.8903 ∼ 0.8905	145.30 ∼146.51	−0.90
150.00	0.8522 ∼ 0.8526	152.51 ∼ 154.49	−3.50

**Table 4.** XML message format for data transmission.

**Tags**	**Description**
<message>	Start protocol messages
<header>	Messages
<body>	When you request—Setting the argument to the command
	When you response—Save measurement data value
<type>	When you request—“request”
	When you response—“response”
<src>	Message sender ID
<tar>	Message recipients ID
<command>	Request command
<request>	When asked to respond = 1
	When you do not need a response = 0
<posN>	Measured data value (N = Number of measurements)

**Table 5.** Data samples measured by the proposed measurement method (rotary-type).

**ID**	**Plant name**	**Points (130 points, measured radius of object plant, 2.5° interval, cm)**	**Ave. cross section area (cm^2^)**	**Avg. radius (cm)**	**Circum. (cm)**
1	Plant-1	7.835,7.835,7.815,7.840,7.920,7.925,8.031,8.000,8.038,8.019,8.113,8.150,8.150,8.131, 8.131,8.256,8.262,8.238,8.256,8.250,8.356,8.363,8.381,8.363,8.506,8.500,8.494,8.462, 8.462,8.613,8.594,8.600,8.594,8.719,8.719,8.706,8.719,8.850,8.806,8.825,8.856,8.825, 8.938,8.969,8.944,8.938,8.938,9.107,9.100,9.080,9.087,9.093,9.193,9.213,9.220,9.220, 9.207,9.333,9.327,9.313,9.333,9.347,9.333,9.447,9.467,9.473,9.447,9.467,9.593,9.560, 9.593,9.600,9.567,9.567,9.600,9.733,9.700,9.693,9.713,9.713,9.720,9.713,9.713,9.687, 9.727,9.860,9.827,9.833,9.847,9.867,9.820,9.833,9.973,9.960,9.967,9.980,9.980,9.953, 9.953,9.987,10.165,10.118,10.188,10.165,10.141,10.141,10.165,10.188,10.141,10.165, 10.176,10.141,10.129,10.176,10.353,10.341,10.400,10.376,10.341,10.376,10.412,10.353, 10.353,10.388,10.376,10.388,10.341,10.388,10.588,10.553,10.576,10.600,10.553,10.553, 10.612,10.576,10.588,10.624,10.612,10.565,10.600,10.588,10.588,10.576,10.588,10.612, 10.600,10.788,10.824,10.800,10.812,10.824,10.835,10.788,10.776,10.812,10.812,10.788, 10.847,10.859,10.788,10.765,10.812,10.800,10.753,10.824,10.800,10.788,10.776,10.835, 10.788,10.788,10.800,10.824,10.788,10.765,10.824,10.812,10.765,10.812,10.847,10.812, 10.800,10.835,10.847,10.776,10.824,10.800,10.788,10.776,10.835,10.812,10.788,10.824, 10.835,10.800,10.788,10.835,10.824,10.812,10.824,10.859,10.800,10.788,10.812,10.812, 10.812,10.835,10.824,10.812,10.788,10.812,10.812,10.576,10.624,10.624,10.588,10.576, 10.588,10.600,10.588,10.612,10.600,10.553,10.565,10.365,10.412,10.353,10.365,10.400, 10.353,10.318,10.353,10.365,10.329,10.412,10.306,10.129,10.118,10.153,10.165,10.129, 10.176,10.141,10.106,9.947,9.980,9.967,9.940,9.960,9.960,9.953,9.960,9.847,9.840, 9.827,9.847,9.867,9.833,9.813,9.867,9.840,9.707,9.713,9.713,9.693,9.693,9.707,9.733, 9.680,9.600,9.587,9.607,9.593,9.607,9.613,9.573,9.593,9.600,9.447,9.447,9.493,9.473, 9.447,9.473,9.440,9.333,9.347,9.333,9.360,9.327,9.220,9.213,9.193,9.213,9.227,9.220, 9.120,9.087,9.093,9.100,9.093,8.950,8.950,8.950,8.938,8.944,8.794,8.831,8.831,8.831, 8.700,8.706,8.725,8.706,8.569,8.594,8.600,8.587,8.619,8.481,8.469,8.481,8.494,8.500, 8.350,8.375,8.381,8.381,8.231,8.256,8.256,8.244,8.144,8.150,8.125,8.012,8.019,8.056, 7.925,7.925,7.935,7.930,7.825,7.830,7.840,7.820,7.835,7.840,7.740,7.750,7.740,7.735, 7.740,7.750,7.750,7.735,7.715,7.745,7.745,7.720,7.740,7.655,7.635,7.635,7.655,7.675, 7.645,7.565,7.565,7.545,7.545,7.570,7.470,7.445,7.475,7.475,7.475,7.450,7.475,7.380, 7.355,7.380,7.375,7.355,7.365,7.375,7.465,7.455,7.465,7.475,7.465,7.445,7.560,7.570, 7.555,7.560,7.56	234.61	8.64	54.30

2	Plant-1	7.715,7.810,7.825,7.845,7.915,7.920,8.019,8.019,8.000,8.025,8.131,8.137,8.137,8.156, 8.250,8.250,8.250,8.250,8.231,8.262,8.331,8.337,8.356,8.381,8.375,8.462,8.494,8.488, 8.475,8.587,8.606,8.587,8.594,8.719,8.725,8.719,8.712,8.719,8.819,8.812,8.844,8.844, 8.812,8.938,8.956,8.938,8.944,9.080,9.107,9.087,9.093,9.107,9.213,9.187,9.200,9.213, 9.207,9.333,9.347,9.320,9.327,9.333,9.487,9.440,9.473,9.493,9.473,9.467,9.467,9.587, 9.580,9.607,9.607,9.580,9.593,9.593,9.733,9.700,9.727,9.713,9.720,9.687,9.720,9.720, 9.713,9.847,9.873,9.820,9.820,9.840,9.847,9.807,9.973,9.967,9.947,9.973,9.967,9.973, 9.960,9.973,9.973,10.153,10.153,10.153,10.129,10.129,10.188,10.188,10.129,10.165, 10.176,10.129,10.129,10.176,10.388,10.353,10.412,10.376,10.318,10.341,10.400,10.353, 10.341,10.365,10.388,10.388,10.353,10.400,10.400,10.588,10.612,10.600,10.588,10.541, 10.612,10.600,10.553,10.624,10.600,10.576,10.565,10.600,10.588,10.565,10.576,10.624, 10.565,10.788,10.624,10.788,10.812,10.847,10.824,10.800,10.800,10.847,10.800,10.800, 10.824,10.847,10.788,10.776,10.824,10.776,10.788,10.835,10.824,10.800,10.788,10.835, 10.824,10.800,10.835,10.800,10.800,10.800,10.824,10.812,10.800,10.824,10.824,10.776, 10.812,10.824,10.788,10.788,10.835,10.835,10.788,10.847,10.847,10.800,10.776,10.835, 10.847,10.788,10.824,10.812,10.800,10.788,10.824,10.812,10.800,10.835,10.835,10.812, 10.788,10.835,10.812,10.788,10.812,10.847,10.765,10.824,10.576,10.588,10.565,10.612, 10.612,10.576,10.576,10.600,10.635,10.565,10.624,10.647,10.565,10.376,10.376,10.353, 10.329,10.412,10.388,10.412,10.388,10.388,10.388,10.129,10.188,10.153,10.153,10.165, 10.176,10.106,10.129,10.165,9.973,9.953,9.967,9.987,9.953,9.947,9.967,9.960,9.827, 9.860,9.840,9.847,9.813,9.847,9.827,9.820,9.853,9.713,9.700,9.700,9.727,9.720,9.700, 9.727,9.700,9.560,9.587,9.593,9.593,9.593,9.600,9.600,9.440,9.580,9.473,9.460,9.433, 9.480,9.487,9.447,9.467,9.333,9.340,9.333,9.340,9.347,9.333,9.220,9.213,9.193,9.207, 9.213,9.107,9.073,9.080,9.100,9.080,8.931,8.962,8.950,8.925,8.938,8.875,8.806,8.812, 8.831,8.831,8.706,8.719,8.719,8.719,8.581,8.606,8.587,8.606,8.494,8.512,8.469,8.481, 8.494,8.363,8.350,8.363,8.381,8.238,8.250,8.269,8.137,8.125,8.144,8.150,8.012,8.025, 8.038,7.915,7.915,7.935,7.930,7.830,7.840,7.820,7.835,7.820,7.845,7.740,7.735,7.745, 7.750,7.740,7.725,7.750,7.750,7.725,7.740,7.745,7.735,7.740,7.645,7.640,7.645,7.655, 7.665,7.640,7.550,7.565,7.565,7.550,7.560,7.480,7.465,7.460,7.460,7.455,7.460,7.465, 7.445,7.365,7.365,7.365,7.360,7.360,7.470,7.475,7.455,7.460,7.465,7.460,7.455,7.560, 7.570,7.550,7.565,7.55	235.21	8.65	54.37

3	Plant-1	7.835,7.840,7.810,7.825,7.940,7.925,7.905,8.044,8.038,8.019,8.125,8.131,8.125,8.131, 8.137,8.231,8.250,8.231,8.238,8.262,8.231,8.375,8.387,8.337,8.363,8.500,8.475,8.462, 8.488,8.613,8.594,8.587,8.619,8.725,8.719,8.719,8.719,8.712,8.825,8.837,8.806,8.812, 8.844,8.956,8.931,8.956,8.950,9.093,9.073,9.100,9.100,9.073,9.220,9.227,9.207,9.220, 9.227,9.340,9.340,9.340,9.367,9.347,9.320,9.480,9.467,9.453,9.487,9.460,9.460,9.567, 9.587,9.580,9.593,9.587,9.600,9.587,9.707,9.727,9.693,9.700,9.720,9.727,9.700,9.707, 9.727,9.693,9.813,9.847,9.867,9.840,9.847,9.847,9.947,9.960,9.973,9.960,9.940,9.953, 9.960,9.967,9.927,10.165,10.200,10.129,10.153,10.153,10.141,10.141,10.165,10.165, 10.153,10.141,10.153,10.141,10.318,10.365,10.365,10.341,10.376,10.400,10.353,10.329, 10.388,10.365,10.353,10.388,10.412,10.341,10.576,10.624,10.565,10.576,10.612,10.612, 10.588,10.565,10.612,10.553,10.565,10.612,10.588,10.576,10.588,10.612,10.600,10.788, 10.635,10.812,10.576,10.588,10.835,10.800,10.776,10.824,10.847,10.812,10.824,10.824, 10.835,10.800,10.835,10.800,10.788,10.824,10.847,10.765,10.788,10.824,10.824,10.788, 10.812,10.835,10.812,10.812,10.812,10.812,10.776,10.835,10.812,10.812,10.776,10.824, 10.835,10.800,10.788,10.835,10.835,10.788,10.824,10.824,10.765,10.835,10.835,10.776, 10.812,10.812,10.800,10.765,10.824,10.812,10.788,10.835,10.824,10.800,10.788,10.812, 10.847,10.788,10.800,10.812,10.788,10.812,10.847,10.600,10.565,10.588,10.588,10.529, 10.565,10.588,10.612,10.565,10.624,10.624,10.588,10.588,10.635,10.365,10.365,10.376, 10.376,10.365,10.400,10.341,10.388,10.388,10.353,10.141,10.153,10.129,10.094,10.165, 10.165,10.141,9.973,9.987,9.960,9.947,9.980,9.993,9.927,9.967,9.960,9.947,9.807,9.847, 9.847,9.827,9.847,9.853,9.820,9.833,9.727,9.720,9.733,9.733,9.727,9.720,9.707,9.740, 9.713,9.573,9.587,9.600,9.587,9.600,9.593,9.587,9.593,9.593,9.473,9.440,9.460,9.480, 9.460,9.440,9.473,9.360,9.333,9.347,9.340,9.320,9.327,9.220,9.240,9.207,9.220,9.213, 9.073,9.073,9.080,9.113,9.040,8.938,8.925,8.944,8.919,8.931,8.831,8.856,8.819,8.819, 8.725,8.700,8.700,8.719,8.613,8.600,8.587,8.594,8.587,8.469,8.494,8.500,8.475,8.363, 8.375,8.350,8.369,8.256,8.262,8.256,8.250,8.156,8.125,8.113,8.150,8.019,8.006,8.012, 7.930,7.920,7.910,7.925,7.930,7.825,7.845,7.845,7.830,7.730,7.750,7.745,7.740,7.740, 7.755,7.725,7.735,7.745,7.730,7.735,7.745,7.755,7.750,7.630,7.660,7.660,7.640,7.660, 7.565,7.545,7.555,7.545,7.565,7.565,7.450,7.470,7.470,7.455,7.465,7.480,7.460,7.355, 7.385,7.385,7.365,7.370,7.370,7.365,7.445,7.470,7.465,7.455,7.455,7.475,7.465,7.540, 7.560,7.565,7.54	235.44	8.66	54.39

**Table 6.** Comparisons of measurement results.

**(a)** Direct measurement and fixed-type measurement.											

**Measurement period**		**1**	**2**	**3**	**4**	**5**	**6**	**7**	**8**	**9**	**10**
Direct-type	Circumference (mm)	263.0	265.0	267.0	2730	277.0	281.0	284.0	290.0	293.0	295.0
	Cross sectional area (mm^2^)	5,504.3	5,588.3	5,673.0	5,930.8	6,105.9	6,283.5	6,418.4	6,692.5	6,831.6	6,925.2

Fixed-type	Circumference (mm)	257.4	259.1	263.9	266.2	270.9	274.6	277.1	282.2	285.7	288.3
	Cross sectional area (mm^2^)	5,272.4	5,342.3	5,542.0	5,639.1	5,839.9	6,000.6	6,110.3	6,337.3	6,495.5	6,614.2
Circumference Error		−5.6	−5.9	−3.1	−6.8	−6.1	−6.4	−6.9	−7.8	−7.3	−6.7

Circumference Error Rate (%)		−2.2	−2.3	−1.2	−2.6	−2.3	−2.3	−2.5	−2.8	−2.6	−2.3

**(b)** Direct measurement and rotary-type measurement.											

**Measurement period**		**1**	**2**	**3**	**4**	**5**	**6**	**7**	**8**	**9**	**10**
Direct-type	Circumference (mm)	238.0	240.0	242.0	248.0	252.0	256.0	259.0	264.0	267.0	269.0
	Cross sectional area (mm^2^)	4,507.6	4,583.7	4,660.4	4,894.3	5,053.5	5,215.2	5,338.1	5,546.2	5,673.0	5,758.3

Fixed-type	Circumference (mm)	234.4	235.1	237.7	241.8	246.5	249.3	253.9	259.8	262.4	265.7
	Cross sectional area (mm^2^)	4,372.3	4,398.4	4,496.2	4,652.7	4,835.3	4,945.8	5,130.0	5,371.2	5,479.2	5,617.9
Circumference Error		−3.6	−4.9	−4.3	−6.2	−5.5	−6.7	−5.1	−4.2	−4.6	−3.3

Circumference Error Rate (%)		−1.5	−2.1	−1.8	−2.6	−2.2	−2.7	−2.0	−1.6	−1.8	−1.2

**Table 7.** Qualitative comparison and analysis of measurement methods.

**Qualitative performance items**	**Existing Methods**		**Proposed Methods**	

	**Direct-type**	**Contact-type**	**Fixed-type**	**Rotary-type**
Measurement of Irregular Surface	Impossible	Impossible	Partially	Available
Remote Measurement & Control	Impossible	Partially	Available	Available
Real-time Measurement	Impossible	Partially	Available	Available
Measuring Cost	High	Middle	Low	Low
Measurement error	Critical	Moderate	Moderate	Tolerable
Thickening Form Measurement	Impossible	Impossible	Available	Available
Initial Installation Costs	Low	High	High	High
Damage to the Plant in Measurement	Much	Moderate	Negligible	Negligible
System Operating Cost	Low	High	High	High
Data Analysis and Provisioning	Partially	Partially	Available	Available
